# Supplementary material for: Analysis of mobility level of COVID-19 patients undergoing mechanical ventilation support: A single center, retrospective cohort study
Source: PLoS One. 2022 Aug 1;17(8):e0272373. doi: 10.1371/journal.pone.0272373 (PMC9342786; doi:10.1371/journal.pone.0272373)
Supplement: S1 Table — Definition of abbreviations: SAPS: simplified acute physiology score; SOFA = sequential organ failure assessment; ICU = intensive care unit; COPD = chronic obstructive pulmonary disease; PaO2 = partial pressure of oxygen; FiO2 = fraction of inspired oxygen; PaCO2 = partial pressure of carbon dioxide; ECMO = extracorporeal membrane oxygenation. (DOCX) [file pone.0272373.s001.docx]

| **S1 Table –** Rate of Missing Data | |
| --- | --- |
|  | **Overall (*n*=949)** |
| Age | 0 (0) |
| Gender | 0 (0) |
| BMI – no., % | 181 (19.1) |
| SAPS III | 0 (0) |
| SOFA | 0 (0) |
| Hours between hospital and ICU admission | 0 (0) |
| Charlson comorbidity score | 0 (0) |
| Modified frailty score | 0 (0) |
| Readmission | 0 (0) |
| Co-morbidities – no., % |  |
| Chronic kidney disease | 190 (20) |
| COPD | 190 (20) |
| Cirrhosis | 190 (20) |
| Diabetes | 190 (20) |
| Heart failure | 190 (20) |
| Hematological cancer | 190 (20) |
| Hemodialysis | 190 (20) |
| Immunosuppression | 190 (20) |
| Metastatic | 190 (20) |
| Previous myocardial infarction | 190 (20) |
| Solid neoplasia | 190 (20) |
| Within the first hour of ICU admission |  |
| Non-invasive ventilation | 0 (0) |
| Invasive mechanical ventilation | 0 (0) |
| Renal replacement therapy | 0 (0) |
| Vasopressor | 0 (0) |
| Acute kidney injury | 0 (0) |
| Limitation of treatment | 0 (0) |
| Vital signs within 24 hours of admission – no., % |  |
| Highest temperature | 86 (9.1) |
| Lowest mean arterial pressure | 73 (7.7) |
| Highest heart rate | 100 (10.5) |
| Pathology within 24 hours of admission – no., % |  |
| Highest white blood cell count | 635 (66.9) |
| Lowest platelet | 632 (66.6) |
| Highest creatinine | 646 (68.1) |
| pH | 748 (78.8) |
| PaO_2_ / FiO_2_ | 750 (79) |
| PaCO_2_ | 748 (78.8) |
| Lactate | 749 (78.9) |
| Within 24 hours of ICU admission – no., % |  |
| Invasive mechanical ventilation | 0 (0) |
| Non-invasive ventilation | 0 (0) |
| Vasopressor | 0 (0) |
| Acute kidney injury | 0 (0) |
| Renal replacement therapy | 0 (0) |
| During ICU stay – no., % |  |
| Non-invasive ventilation | 0 (0) |
| Invasive mechanical ventilation | 0 (0) |
| Tracheostomy | 0 (0) |
| High-flow nasal canula | 0 (0) |
| Vasopressor | 0 (0) |
| Renal replacement therapy | 0 (0) |
| ECMO | 0 (0) |
| Use of neuromuscular blocking agent | 0 (0) |
| Perme score at admission | 0 (0) |
| Duration of ventilation – no., % | 53 (13.4) |
| ICU length of stay | 0 (0) |
| Hospital length of stay – no., % | 11 (1.2) |
| ICU mortality | 0 (0) |
| Hospital mortality – no., % | 11 (1.2) |
| 28-day mortality | 0 (0) |

Definition of abbreviations: SAPS: simplified acute physiology score; SOFA = sequential organ failure assessment; ICU = intensive care unit; COPD = chronic obstructive pulmonary disease; PaO_2_ = partial pressure of oxygen; FiO_2_ = fraction of inspired oxygen; PaCO_2_ = partial pressure of carbon dioxide; ECMO = extracorporeal membrane oxygenation.
