# Supplementary material for: Analysis of mobility level of COVID-19 patients undergoing mechanical ventilation support: A single center, retrospective cohort study
Source: PLoS One. 2022 Aug 1;17(8):e0272373. doi: 10.1371/journal.pone.0272373 (PMC9342786; doi:10.1371/journal.pone.0272373)
Supplement: S2 Table — Data are median and interquartile range (quartile 25%—quartile 75%) or n (%). Percentages may not total 100 because of rounding. Definition of abbreviations: PMI = perme mobility index; ICU = intensive care unit; NMBA = neuromuscular blockade; ECMO = extracorporeal membrane oxygenation. *The Perme Mobility Index (PMI) is calculated by the difference between the total Perme Score at ICU discharge and the total Perme Score at ICU admission, divided by the ICU length of stay (ICU LOS) [PMI = ΔPerme Score (ICU discharge–ICU admission) / ICU LOS]. The result is a dimensionless number and it can be either positive or negative. Positive values are associated with patients that improve the mobility status during ICU stay, whereas negative values are associated with patients that decrease mobility status during ICU stay. (DOCX) [file pone.0272373.s002.docx]

| **S2 Table –** Organ Support During ICU Stay | | | | | | | |
| --- | --- | --- | --- | --- | --- | --- | --- |
|  |  | **Mechanical Ventilation (*n*=396)** | | | **No Mechanical Ventilation (*n*=553)** | | |
|  | **Overall**  **(*n*=949)** | **Improved PMI***  **(*n*=202)** | **Not Improved PMI**  **(*n*=194)** | ***p* value** | **Improved PMI**  **(*n*=322)** | **Not Improved PMI**  **(*n*=231)** | ***p* value** |
| Within 24 hours of ICU admission – no. (%) |  |  |  |  |  |  |  |
| Invasive mechanical ventilation | 153 (16.1) | 105 (52) | 48 (24.7) | <0.001 | 0 (0) | 0 (0) | – |
| Non-invasive ventilation | 417 (43.9) | 96 (47.5) | 95 (49) | 0.84 | 156 (48.4) | 70 (30.3) | <0.001 |
| Vasopressor | 150 (15.8) | 93 (46) | 50 (25.8) | <0.001 | 4 (1.2) | 3 (1.3) | 0.99 |
| Acute kidney injury | 58 (6.1) | 20 (9.9) | 24 (12.4) | 0.52 | 6 (1.9) | 8 (3.5) | 0.27 |
| Renal replacement therapy | 13 (1.4) | 8 (4) | 4 (2.1) | 0.38 | 0 (0) | 1 (0.4) | 0.41 |
| During ICU stay – no. (%) |  |  |  |  |  |  |  |
| Non-invasive ventilation | 617 (65) | 171 (84.7) | 142 (73.2) | 0.006 | 199 (61.8) | 105 (45.5) | <0.001 |
| Invasive mechanical ventilation | 396 (41.7) | 202 (100) | 194 (100) | – | 0 (0) | 0 (0) | – |
| Tracheostomy | 76 (8) | 31 (15.3) | 43 (22.2) | 0.09 | 0 (0) | 2 (0.9) | 0.17 |
| High-flow nasal canula | 355 (37.4) | 81 (40.1) | 96 (49.5) | 0.06 | 123 (38.2) | 55 (23.8) | <0.001 |
| Renal replacement therapy | 137 (14.4) | 43 (21.3) | 91 (46.9) | <0.001 | 0 (0) | 3 (1.3) | 0.07 |
| ECMO | 17 (1.8) | 4 (2) | 13 (6.7) | 0.02 | 0 (0) | 0 (0) | – |
| Use of vasopressor | 380 (40) | 188 (93.1) | 175 (90.2) | 0.36 | 10 (3.1) | 7 (3) | 0.99 |
| Use of NMBA | 304 (32) | 139 (68.8) | 165 (85.1) | <0.001 | 0 (0) | 0 (0) | – |
| Data are median and interquartile range (quartile 25% - quartile 75%) or n (%). Percentages may not total 100 because of rounding.  *Definition of abbreviations:* PMI = perme mobility index; ICU = intensive care unit; NMBA = neuromuscular blockade; ECMO = extracorporeal membrane oxygenation.  *The Perme Mobility Index (PMI) is calculated by the difference between the total Perme Score at ICU discharge and the total Perme Score at ICU admission, divided by the ICU length of stay (ICU LOS) [PMI = ΔPerme Score (*ICU discharge* – *ICU admission*) / ICU LOS]. The result is a dimensionless number and it can be either positive or negative. Positive values are associated with patients that improve the mobility status during ICU stay, whereas negative values are associated with patients that decrease mobility status during ICU stay. | | | | | | | |
