# Supplementary material for: Analysis of mobility level of COVID-19 patients undergoing mechanical ventilation support: A single center, retrospective cohort study
Source: PLoS One. 2022 Aug 1;17(8):e0272373. doi: 10.1371/journal.pone.0272373 (PMC9342786; doi:10.1371/journal.pone.0272373)
Supplement: S3 Table — Data are median and interquartile range (quartile 25%—quartile 75%) or n (%). Percentages may not total 100 because of rounding. Definition of abbreviations: PMI = perme mobility index; ICU = intensive care unit. *The Perme Mobility Index (PMI) is calculated by the difference between the total Perme Score at ICU discharge and the total Perme Score at ICU admission, divided by the ICU length of stay (ICU LOS) [PMI = Δ Perme Score (ICU discharge–ICU admission) / ICU LOS]. The result is a dimensionless number and it can be either positive or negative. Positive values are associated with patients that improve the mobility status during ICU stay, whereas negative values are associated with patients that decrease mobility status during ICU stay. †Perme ICU mobility score range from 0 to 32, with higher scores indicating better mobility level. (DOCX) [file pone.0272373.s003.docx]

| **S3 Table –** Perme Description in the Included Patients | | | | | | | | |
| --- | --- | --- | --- | --- | --- | --- | --- | --- |
|  |  | **Mechanical Ventilation**  **(*n*=396)** | | | **No Mechanical Ventilation**  **(*n*=553)** | | | |
|  | **Overall**  **(*n*=949)** | **Improved PMI**  **(*n*=202)** | **Not Improved PMI**  **(*n*=194)** | ***p* value** | | **Improved PMI**  **(*n*=322)** | **Not Improved PMI**  **(*n*=231)** | ***p* value** |
| Perme mobility index* | 0.2 (-0.2–1.1) | 0.7 (0.3–1.4) | -0.1 (-0.5–0) | <0.001 | | 1.4 (0.5–2.7) | -1 (-5.3–0) | <0.001 |
| Perme score^†^ |  |  |  |  | |  |  |  |
| Admission | 10 (4–24) | 0 (0–8) | 8 (0–18.8) | <0.001 | | 14 (8–24) | 26 (10–30) | <0.001 |
| Day 3 | 6 (0–19) | 0 (0–2) | 0 (0–8) | 0.15 | | 23 (9–29) | 10 (6–23) | <0.001 |
| Day 5 | 0 (0–9) | 0 (0–1) | 0 (0–0) | 0.09 | | 21 (9–27) | 9 (1.5–26.5) | 0.10 |
| Day 7 | 0 (0–8) | 0 (0–4) | 0 (0–0) | <0.001 | | 23 (9–26) | 8 (6–24) | 0.02 |
| Day 9 | 0 (0–8) | 0 (0–5.5) | 0 (0–0) | <0.001 | | 23 (14.5–28) | 23 (9–26) | 0.48 |
| Day 11 | 0 (0–3) | 0 (0–6) | 0 (0–0) | <0.001 | | 18 (7.8–28) | 9.5 (7.5–18.2) | 0.33 |
| Day 13 | 0 (0–7) | 2.5 (0–8.8) | 0 (0–0) | <0.001 | | 10 (3.2–25.2) | 16.5 (9.5–26.5) | 0.68 |
| Day 15 | 0 (0–6) | 3 (0–12) | 0 (0–0) | <0.001 | | 7 (2–8) | 9.5 (5–25.2) | 0.59 |
| Day 17 | 0 (0–6) | 2 (0–8) | 0 (0–2) | 0.001 | | 6 (2–9) | 3 (2–6) | 0.67 |
| Day 19 | 0 (0–4) | 1 (0–9) | 0 (0–0) | <0.001 | | 2 (1.5–4) | 9 (5.5–9.5) | 0.18 |
| Day 21 | 0 (0–3) | 1 (0–13) | 0 (0–0) | <0.001 | | 19 (19–19) | – | – |
| Day 23 | 0 (0–3) | 2 (0–8.8) | 0 (0–0) | <0.001 | | 7 (5–13.5) | – | – |
| Day 25 | 0 (0–3) | 3 (0–7) | 0 (0–0) | <0.001 | | 3 (3–3) | – | – |
| Day 27 | 0 (0–4) | 1 (0–6) | 0 (0–2.8) | 0.02 | | 15 (10–20) | – | – |
| ICU discharge | 23 (6–29) | 20 (11–26) | 0 (0–3) | <0.001 | | 29 (25–31) | 22 (7.5–29) | <0.001 |
| Data are median and interquartile range (quartile 25% - quartile 75%) or n (%). Percentages may not total 100 because of rounding.  *Definition of abbreviations:* PMI = perme mobility index; ICU = intensive care unit.  *The Perme Mobility Index (PMI) is calculated by the difference between the total Perme Score at ICU discharge and the total Perme Score at ICU admission, divided by the ICU length of stay (ICU LOS) [PMI = ΔPerme Score (*ICU discharge* – *ICU admission*) / ICU LOS]. The result is a dimensionless number and it can be either positive or negative. Positive values are associated with patients that improve the mobility status during ICU stay, whereas negative values are associated with patients that decrease mobility status during ICU stay.  ^†^Perme ICU mobility score range from 0 to 32, with higher scores indicating better mobility level. | | | | | | | | |
