# Supplementary material for: Analysis of mobility level of COVID-19 patients undergoing mechanical ventilation support: A single center, retrospective cohort study
Source: PLoS One. 2022 Aug 1;17(8):e0272373. doi: 10.1371/journal.pone.0272373 (PMC9342786; doi:10.1371/journal.pone.0272373)
Supplement: S4 Table — Definition of abbreviations: GVIF = generalized variance-inflation factor; df = degrees of freedom; SAPS = simplified acute physiology score; SOFA = Sequential Organ Failure Assessment; ECMO = extracorporeal membrane oxygenation; NMBA = neuromuscular blockade; ICU = intensive care unit. (DOCX) [file pone.0272373.s004.docx]

| **S4 Table -** Multicollinearity and Linearity Assumption in the Final Model | | | |
| --- | --- | --- | --- |
|  | **GVIF** | **GVIF^1/(2-df)^** | **Box-Tidwell *p* value** |
| Age | 2.367473 | 1.538659 | 0.84 |
| SAPS III | 3.201718 | 1.789334 | 0.55 |
| SOFA | 3.614804 | 1.901264 | 0.03 |
| Charlson co-morbidity index | 1.463860 | 1.209901 | 0.20 |
| Modified frailty score | 1.687305 | 1.139721 | – |
| Palliative care | 1.111870 | 1.054453 | – |
| Non-invasive ventilation | 1.285914 | 1.064886 | – |
| Invasive mechanical ventilation | 15.338907 | 1.979013 | – |
| Vasopressor | 11.334755 | 1.834860 | – |
| Tracheostomy | 1.328604 | 1.152651 | – |
| Renal replacement therapy | 1.567264 | 1.251904 | – |
| ECMO | 1.087218 | 1.042698 | – |
| Use of NMBA | 3.100954 | 1.760953 | – |
| Perme score at admission | 1.955494 | 1.398390 | 0.59 |
| ICU length of stay | 1.438760 | 1.199483 | 0.09 |
| *Definition of abbreviations:* GVIF = generalized variance-inflation factor; df = degrees of freedom; SAPS = simplified acute physiology score; SOFA = Sequential Organ Failure Assessment; ECMO = extracorporeal membrane oxygenation; NMBA = neuromuscular blockade; ICU = intensive care unit. | | | |
