# Supplementary material for: Analysis of mobility level of COVID-19 patients undergoing mechanical ventilation support: A single center, retrospective cohort study
Source: PLoS One. 2022 Aug 1;17(8):e0272373. doi: 10.1371/journal.pone.0272373 (PMC9342786; doi:10.1371/journal.pone.0272373)
Supplement: S5 Table — Data are median and interquartile range (quartile 25%—quartile 75%) or n (%). Percentages may not total 100 because of rounding. Definition of abbreviations: ICU = intensive care unit. *Charlson comorbidity index range from 0 to 5 for each comorbidity, with score of zero indicating that no comorbidities were found. The higher the score, the more likely the predicted outcome will result in mortality or higher resource use. †The body-mass index (BMI) is calculated by weight in kilograms divided by the square of the height in meters (Kg/m2). The categories are the same for men and women of all body types and ages, as follows: below 18.5 –underweight, 18.5–24.9 –normal or healthy weight, 25.0–29.9 –overweight, and 30.0 and above–obese. ‡Modified Frailty Index–categorized frailty using MFI values into non-frail (MFI = 0), pre-frail (MFI = 1–2) or frail (MFI ≥ 3). (DOCX) [file pone.0272373.s005.docx]

| **S5 Table -** Degree of Mobilization According to Baseline Status   \|  \| **Charlson Comorbidity Score*** \| \| \| **Body Mass Index**^†^ \| \| \| \| **Modified Frailty Index**^‡^ \| \| \| \| \| --- \| --- \| --- \| --- \| --- \| --- \| --- \| --- \| --- \| --- \| --- \| --- \| \|  \| **≥ 1**  **(*n* = 493)** \| **< 1**  **(*n* = 456)** \| ***p* value** \| **≤ 25**  **(*n* = 198)** \| **25–30**  **(*n* = 332)** \| **> 30**  **(*n* = 238)** \| ***p* value** \| **Non-Frail**  **(*n* = 327)** \| **Pre-Frail**  **(*n* = 462)** \| **Frail**  **(*n* = 160)** \| ***P* value** \| \| At ICU admission \|  \|  \|  \|  \|  \|  \|  \|  \|  \|  \|  \| \| Out of bed – no. (%) \| 178 (36.1) \| 205 (45) \| 0.007 \| 85 (42.9) \| 124 (37.3) \| 84 (35.3) \| 0.24 \| 152 (46.5) \| 180 (39) \| 51 (31.9) \| 0.006 \| \| Walked any distance – no. (%) \| 62 (12.6) \| 118 (25.9) \| <0.001 \| 31 (15.7) \| 63 (19) \| 37 (15.5) \| 0.47 \| 81 (24.8) \| 81 (17.5) \| 18 (11.2) \| 0.001 \| \| 1–15 meters \| 26 (5.3) \| 43 (9.4) \|  \| 12 (6.1) \| 23 (6.9) \| 12 (5) \|  \| 30 (9.2) \| 28 (6.1) \| 11 (6.9) \|  \| \| 15–30 meters \| 10 (2.0) \| 22 (4.8) \| <0.001 \| 3 (1.5) \| 16 (4.8) \| 7 (2.9) \| 0.51 \| 12 (3.7) \| 17 (3.7) \| 3 (1.9) \| 0.001 \| \| > 30 meters \| 26 (5.3) \| 53 (11.6) \|  \| 16 (8.1) \| 24 (7.2) \| 18 (7.6) \|  \| 39 (11.9) \| 36 (7.8) \| 4 (2.5) \|  \| \| During ICU stay \|  \|  \|  \|  \|  \|  \|  \|  \|  \|  \|  \| \| Out of bed – no. (%) \| 247 (50.1) \| 295 (64.7) \| <0.001 \| 112 (56.6) \| 177 (53.3) \| 139 (58.4) \| 0.46 \| 215 (65.7) \| 261 (56.5) \| 66 (41.2) \| <0.001 \| \| Days until first occurrence \| 0 (0–3) \| 0 (0–3) \| 0.57 \| 0 (0–0) \| 0 (0–3) \| 0 (0–5) \| 0.03 \| 0 (0–3) \| 0 (0–3) \| 0 (0–0) \| 0.48 \| \| Walked any distance – no. (%) \| 113 (22.9) \| 168 (36.8) \| <0.001 \| 53 (26.8) \| 91 (27.4) \| 70 (29.4) \| 0.80 \| 120 (36.7) \| 125 (27.1) \| 36 (22.5) \| 0.001 \| \| Days until first occurrence \| 0 (0–5) \| 0 (0–3) \| 0.01 \| 0 (0–3) \| 0 (0–3) \| 0 (0–5) \| 0.21 \| 0 (0–3) \| 0 (0–3) \| 1.5 (0–5) \| 0.18 \| \| Walked 1–15 meters – no. (%) \| 56 (11.4) \| 68 (14.9) \| 0.12 \| 26 (13.1) \| 38 (11.4) \| 27 (11.3) \| 0.80 \| 46 (14.1) \| 54 (11.7) \| 24 (15.0) \| 0.42 \| \| Days until first occurrence \| 3 (0–5) \| 0 (0–3) \| 0.12 \| 3 (0–5) \| 0 (0–3) \| 3 (0–5) \| 0.36 \| 0 (0–3) \| 0 (0–5) \| 3 (0–5) \| 0.53 \| \| Walked 15–30 meters – no. (%) \| 25 (5.1) \| 47 (10.3) \| 0.003 \| 10 (5.1) \| 31 (9.3) \| 18 (7.6) \| 0.20 \| 30 (9.2) \| 33 (7.1) \| 9 (5.6) \| 0.35 \| \| Days until first occurrence \| 3 (0–11) \| 3 (0–4) \| 0.20 \| 3 (1–21) \| 0 (0–8) \| 3 (0–8) \| 0.43 \| 3 (0–6) \| 0 (0–5) \| 3 (0–11) \| 0.47 \| \| Walked > 30 meters – no. (%) \| 46 (9.3) \| 78 (17.1) \| <0.001 \| 26 (13.1) \| 38 (11.4) \| 31 (13) \| 0.78 \| 59 (18) \| 58 (12.6) \| 7 (4.4) \| <0.001 \| \| Days until first occurrence \| 0 (0–5) \| 0 (0–4) \| 0.30 \| 0 (0–5) \| 0 (0–5) \| 0 (0–4) \| 0.98 \| 0 (0–5) \| 0 (0–5) \| 0 (0–4) \| 0.91 \| \| At ICU discharge \|  \|  \|  \|  \|  \|  \|  \|  \|  \|  \|  \| \| Out of bed – no. (%) \| 222 (51.2) \| 304 (76.6) \| <0.001 \| 104 (61.5) \| 185 (62.3) \| 138 (65.1) \| 0.74 \| 222 (79) \| 246 (59.9) \| 58 (41.7) \| <0.001 \| \| Walked any distance – no. (%) \| 136 (31.3) \| 199 (50.1) \| <0.001 \| 66 (39.1) \| 116 (39.1) \| 81 (38.2) \| 0.97 \| 143 (50.9) \| 163 (39.7) \| 29 (20.9) \| <0.001 \| \| 1–15 meters \| 43 (9.9) \| 48 (12.1) \|  \| 20 (11.8) \| 34 (11.4) \| 17 (8) \|  \| 34 (12.1) \| 45 (10.9) \| 12 (8.6) \|  \| \| 15–30 meters \| 32 (7.4) \| 42 (10.6) \| <0.001 \| 14 (8.3) \| 22 (7.4) \| 23 (10.8) \| 0.71 \| 24 (8.5) \| 42 (10.2) \| 8 (5.8) \| <0.001 \| \| > 30 meters \| 61 (14.1) \| 109 (27.5) \|  \| 32 (18.9) \| 60 (20.2) \| 41 (19.3) \|  \| 85 (30.2) \| 76 (18.5) \| 9 (6.5) \|  \| \| Data are median and interquartile range (quartile 25% - quartile 75%) or n (%). Percentages may not total 100 because of rounding.  *Definition of abbreviations:* ICU = intensive care unit.  *Charlson comorbidity index range from 0 to 5 for each comorbidity, with score of zero indicating that no comorbidities were found. The higher the score, the more likely the predicted outcome will result in mortality or higher resource use.  ^†^The body-mass index (BMI) is calculated by weight in kilograms divided by the square of the height in meters (Kg/m^2^). The categories are the same for men and women of all body types and ages, as follows: below 18.5 – underweight, 18.5-24.9 – normal or healthy weight, 25.0-29.9 – overweight, and 30.0 and above – obese.  ^‡^Modified Frailty Index – categorized frailty using MFI values into non-frail (MFI = 0), pre-frail (MFI = 1–2) or frail (MFI ≥ 3). \| \| \| \| \| \| \| \| \| \| \| \| |
| --- | --- | --- | --- | --- | --- | --- | --- | --- | --- | --- | --- | --- | --- | --- | --- | --- | --- | --- | --- | --- | --- | --- | --- | --- | --- | --- | --- | --- | --- | --- | --- | --- | --- | --- | --- | --- | --- | --- | --- | --- | --- | --- | --- | --- | --- | --- | --- | --- | --- | --- | --- | --- | --- | --- | --- | --- | --- | --- | --- | --- | --- | --- | --- | --- | --- | --- | --- | --- | --- | --- | --- | --- | --- | --- | --- | --- | --- | --- | --- | --- | --- | --- | --- | --- | --- | --- | --- | --- | --- | --- | --- | --- | --- | --- | --- | --- | --- | --- | --- | --- | --- | --- | --- | --- | --- | --- | --- | --- | --- | --- | --- | --- | --- | --- | --- | --- | --- | --- | --- | --- | --- | --- | --- | --- | --- | --- | --- | --- | --- | --- | --- | --- | --- | --- | --- | --- | --- | --- | --- | --- | --- | --- | --- | --- | --- | --- | --- | --- | --- | --- | --- | --- | --- | --- | --- | --- | --- | --- | --- | --- | --- | --- | --- | --- | --- | --- | --- | --- | --- | --- | --- | --- | --- | --- | --- | --- | --- | --- | --- | --- | --- | --- | --- | --- | --- | --- | --- | --- | --- | --- | --- | --- | --- | --- | --- | --- | --- | --- | --- | --- | --- | --- | --- | --- | --- | --- | --- | --- | --- | --- | --- | --- | --- | --- | --- | --- | --- | --- | --- | --- | --- | --- | --- | --- | --- | --- | --- | --- | --- | --- | --- | --- | --- | --- | --- | --- | --- | --- | --- | --- | --- | --- | --- | --- | --- | --- | --- | --- | --- | --- | --- | --- | --- | --- | --- | --- | --- | --- | --- | --- | --- | --- | --- | --- | --- | --- | --- | --- | --- | --- | --- | --- | --- | --- | --- | --- | --- | --- | --- | --- | --- | --- | --- | --- | --- | --- | --- | --- | --- | --- | --- | --- | --- | --- | --- | --- | --- | --- | --- | --- | --- | --- | --- | --- | --- | --- | --- | --- | --- | --- | --- | --- |
