# Supplementary material for: Analysis of mobility level of COVID-19 patients undergoing mechanical ventilation support: A single center, retrospective cohort study
Source: PLoS One. 2022 Aug 1;17(8):e0272373. doi: 10.1371/journal.pone.0272373 (PMC9342786; doi:10.1371/journal.pone.0272373)
Supplement: S1 Fig — Boxes represent median and interquartile range. Whiskers extend 1.5 times the interquartile range beyond the first and third quartiles per the conventional Tukey method. Transparent circles beyond the whiskers represent outliers. Filled circles represent mean values. *Perme ICU mobility score range from 0 to 32, with higher scores indicating better mobility level. (DOCX) [file pone.0272373.s006.docx]

**S1 Fig –** Perme Score in the First Five Days and at Discharge

**
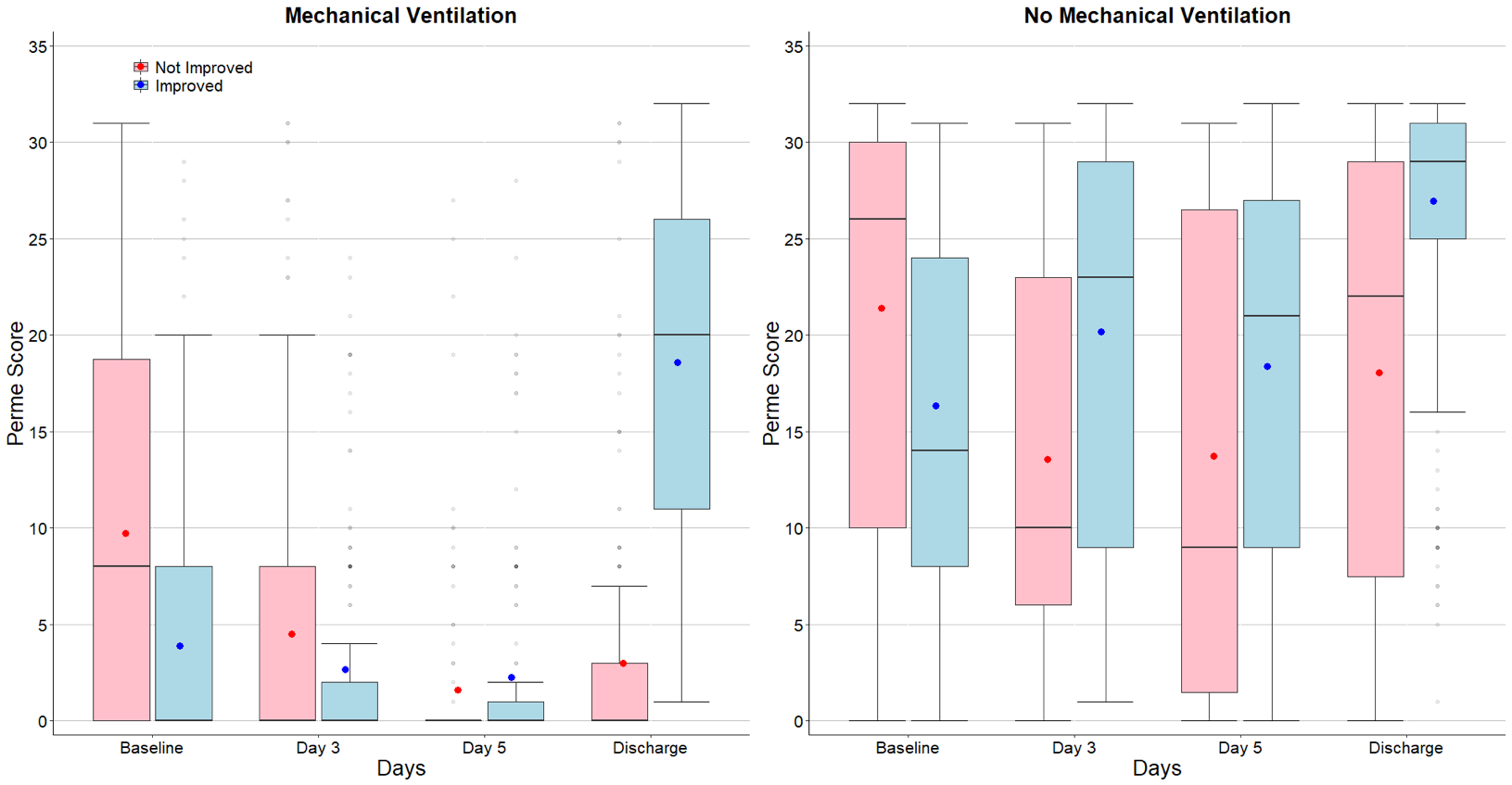
**

Boxes represent median and interquartile range. Whiskers extend 1.5 times the interquartile range beyond the first and third quartiles per the conventional Tukey method. Transparent circles beyond the whiskers represent outliers. Filled circles represent mean values.

*Perme ICU mobility score range from 0 to 32, with higher scores indicating better mobility level.
