# Supplementary material for: Analysis of mobility level of COVID-19 patients undergoing mechanical ventilation support: A single center, retrospective cohort study
Source: PLoS One. 2022 Aug 1;17(8):e0272373. doi: 10.1371/journal.pone.0272373 (PMC9342786; doi:10.1371/journal.pone.0272373)
Supplement: S2 Fig — Definition of abbreviations: ICU = intensive care unit; SAPS III = simplified acute physiology score. The SAPS III score ranges from 0 to 217, with higher scores indicating more severe illness and higher risk of death. (DOCX) [file pone.0272373.s007.docx]

**S2 Fig –** Improvement in mobility over time

**
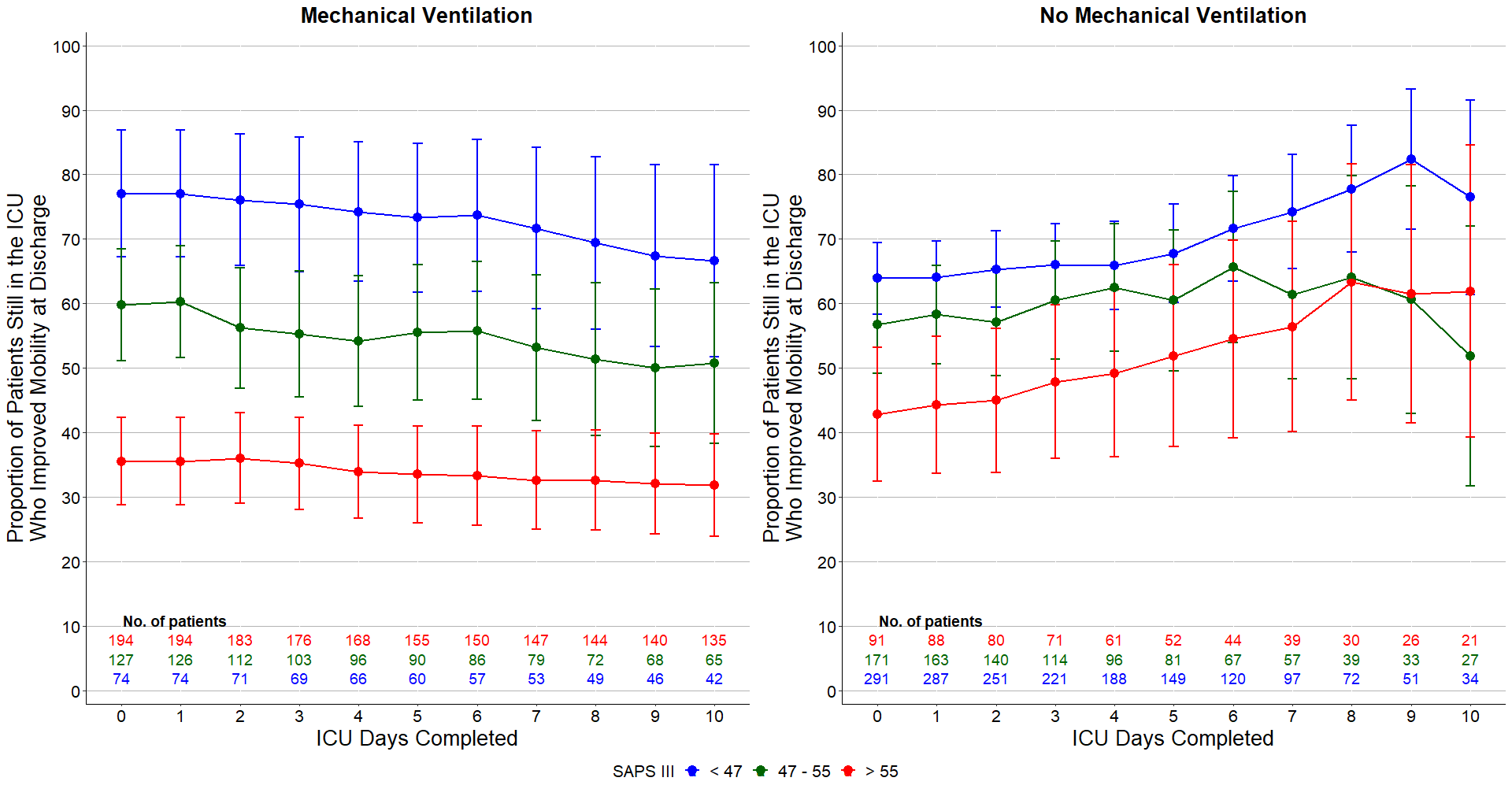
**

*Definition of abbreviations:* ICU = intensive care unit; SAPS III = simplified acute physiology score.

The SAPS III score ranges from 0 to 217, with higher scores indicating more severe illness and higher risk of death.
