# Supplementary material for: Analysis of mobility level of COVID-19 patients undergoing mechanical ventilation support: A single center, retrospective cohort study
Source: PLoS One. 2022 Aug 1;17(8):e0272373. doi: 10.1371/journal.pone.0272373 (PMC9342786; doi:10.1371/journal.pone.0272373)
Supplement: S3 Fig — *Scores on SAPS III range from 0 to 217, with higher scores indicating more severe illness and higher risk of death. †Charlson comorbidity index range from 0 to 5 for each comorbidity, with score of zero indicating that no comorbidities were found. The higher the score, the more likely the predicted outcome will result in mortality or higher resource use. body mass index (BMI) calculated by weight in kilograms divided by the square of the height in meters (Kg/m2), categorized into groups normal or healthy weight (BMI ≤ 25.0), overweight (BMI = 25.0–29.9), and obese (BMI ≥ 30.0). §Modified Frailty Index–categorized frailty using MFI values into non-frail (MFI = 0), pre-frail (MFI = 1–2) or frail (MFI ≥ 3). (DOCX) [file pone.0272373.s008.docx]

**S3 Fig –** Improvement in Mobility Over Time in Specific Subgroups

**
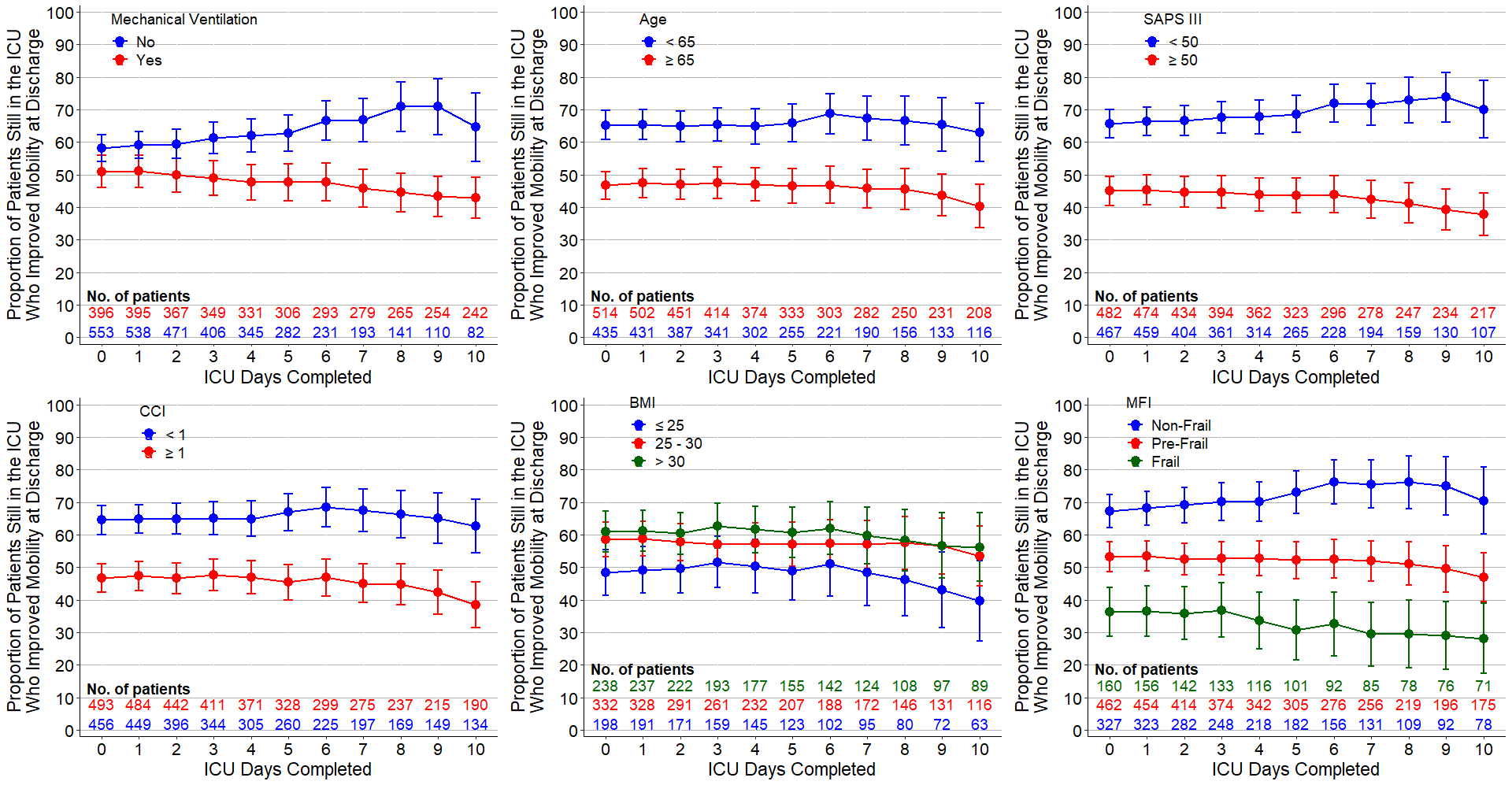
**

*Definition of abbreviations:* SAPS = simplified acute physiology score; CCI = charlson comorbidity index; BMI = body mass index; MFI = modified frailty index; ICU = intensive care unit.

*Scores on SAPS III range from 0 to 217, with higher scores indicating more severe illness and higher risk of death.

^†^Charlson comorbidity index range from 0 to 5 for each comorbidity, with score of zero indicating that no comorbidities were found. The higher the score, the more likely the predicted outcome will result in mortality or higher resource use.

body mass index (BMI) calculated by weight in kilograms divided by the square of the height in meters (Kg/m^2^), categorized into groups normal or healthy weight (BMI ≤ 25.0), overweight (BMI = 25.0-29.9), and obese (BMI ≥ 30.0).

^§^Modified Frailty Index – categorized frailty using MFI values into non-frail (MFI = 0), pre-frail (MFI = 1–2) or frail (MFI ≥ 3).
