# Supplementary material for: Analysis of mobility level of COVID-19 patients undergoing mechanical ventilation support: A single center, retrospective cohort study
Source: PLoS One. 2022 Aug 1;17(8):e0272373. doi: 10.1371/journal.pone.0272373 (PMC9342786; doi:10.1371/journal.pone.0272373)
Supplement: S5 Fig — Unadjusted hazard ratio (HR) calculated with a Cox proportional hazard model. To account for the competing risk of death, patients who died without achieving the event of interest were assigned the worst time possible. a) mechanical ventilation (MV), with groups required (MV = yes) or not required (MV = no); b) simplified acute physiology score (SAPS III score); c) body mass index (BMI) calculated by weight in kilograms divided by the square of the height in meters (Kg/m2), categorized into groups normal or healthy weight (BMI ≤ 25.0), overweight (BMI = 25.0–29.9), and obese (BMI ≥ 30.0); d) age (< 65 or ≥ 65); e) Charlson comorbidity index (CCI) with groups <1 or ≥ 1; and f) Modified Frailty Index (MFI) at the admission, with groups non-frail (MFI = 0), pre-frail (MFI = 1–2) and frail (MFI ≥ 3). (DOCX) [file pone.0272373.s010.docx]

**S5 Fig –** Kaplan-Meier Curves of Time Until the First Walking of More Than 30 Meters

**
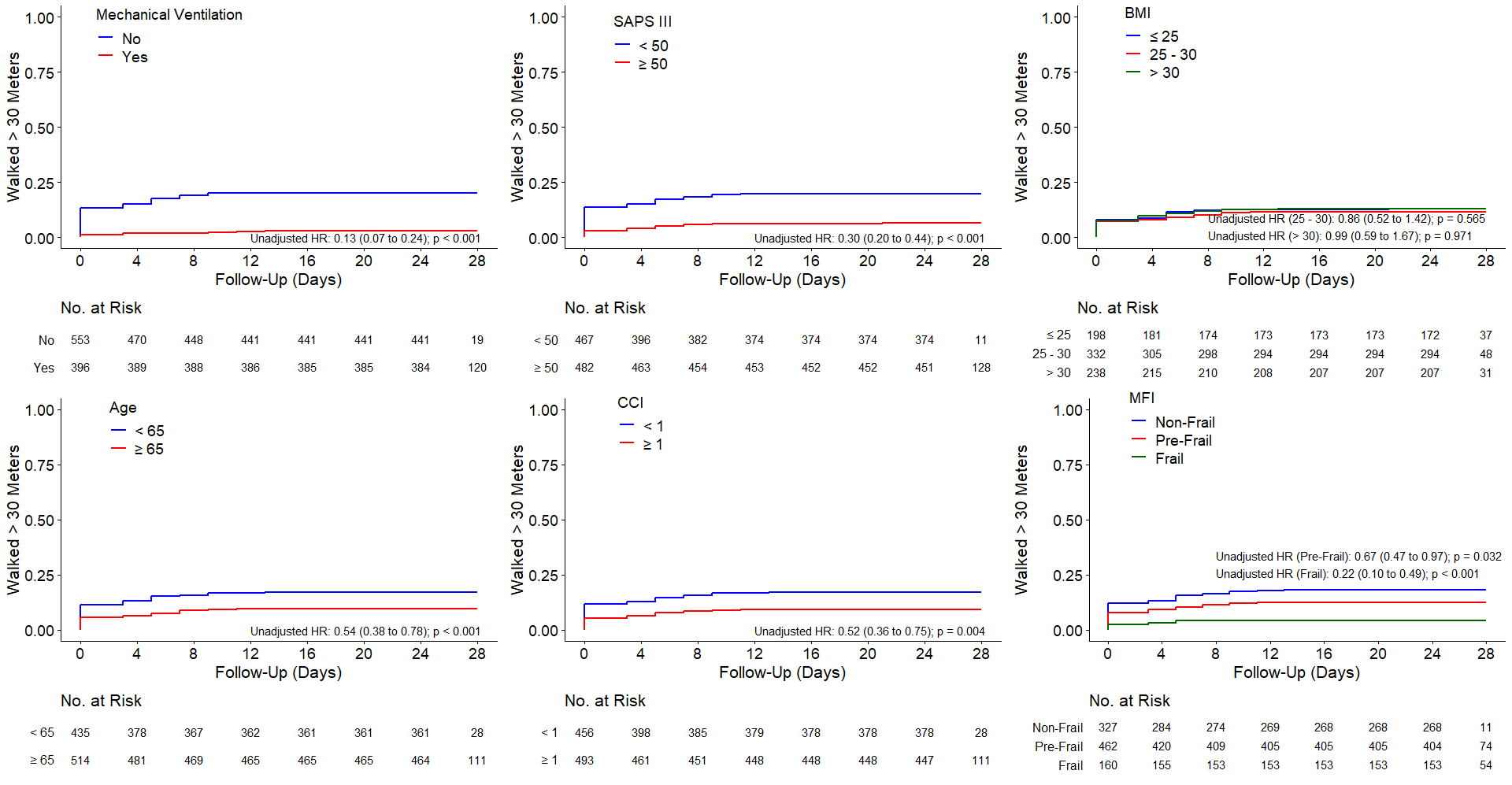
**

Unadjusted hazard ratio (HR) calculated with a Cox proportional hazard model. To account for the competing risk of death, patients who died without achieving the event of interest were assigned the worst time possible. **a)** mechanical ventilation (MV), with groups required (MV = yes) or not required (MV = no) ; **b)** simplified acute physiology score (SAPS III score); **c)** body mass index (BMI) calculated by weight in kilograms divided by the square of the height in meters (Kg/m^2^), categorized into groups normal or healthy weight (BMI ≤ 25.0), overweight (BMI = 25.0-29.9), and obese (BMI ≥ 30.0); **d)** age (< 65 or ≥ 65); **e)** Charlson comorbidity index (CCI) with groups <1 or ≥ 1; and **f)** Modified Frailty Index (MFI) at the admission, with groups non-frail (MFI = 0), pre-frail (MFI = 1–2) and frail (MFI ≥ 3).
